# Malaria Rapid Testing by Community Health Workers Is Effective and Safe for Targeting Malaria Treatment: Randomised Cross-Over Trial in Tanzania

**DOI:** 10.1371/journal.pone.0019753

**Published:** 2011-07-05

**Authors:** Marycelina Mubi, Annika Janson, Marian Warsame, Andreas Mårtensson, Karin Källander, Max G. Petzold, Billy Ngasala, Gloria Maganga, Lars L. Gustafsson, Amos Massele, Göran Tomson, Zul Premji, Anders Björkman

**Affiliations:** 1 Department of Parasitology, School of Public Health, Muhimbili University of Health and Allied Sciences, Dar es Salaam, Tanzania; 2 Unit of Infectious Diseases, Department of Medicine Solna, Karolinska University Hospital/Karolinska Institutet, Stockholm, Sweden; 3 Division of Pediatrics, Department of Clinical Science, Intervention and Technology (CLINTEC), Karolinska Institutet, Stockholm, Sweden; 4 Division of Global Health (IHCAR), Department of Public Health Sciences, Karolinska Institutet, Stockholm, Sweden; 5 Nordic School of Public Health, Gothenburg, Sweden; 6 National Institute of Medical Research, Dar es Salaam, Tanzania; 7 Division of Clinical Pharmacology, Department of Laboratory Medicine, Karolinska Institutet, Stockholm, Sweden; 8 Department of Clinical Pharmacology, School of Medicine, Muhimbili University of Health and Allied Sciences, Dar es Salaam, Tanzania; Mahidol University, Thailand

## Abstract

**Background:**

Early diagnosis and prompt, effective treatment of uncomplicated malaria is critical to prevent severe disease, death and malaria transmission. We assessed the impact of rapid malaria diagnostic tests (RDTs) by community health workers (CHWs) on provision of artemisinin-based combination therapy (ACT) and health outcome in fever patients.

**Methodology/Principal Findings:**

Twenty-two CHWs from five villages in Kibaha District, a high-malaria transmission area in Coast Region, Tanzania, were trained to manage uncomplicated malaria using RDT aided diagnosis or clinical diagnosis (CD) only. Each CHW was randomly assigned to use either RDT or CD the first week and thereafter alternating weekly. Primary outcome was provision of ACT and main secondary outcomes were referral rates and health status by days 3 and 7. The CHWs enrolled 2930 fever patients during five months of whom 1988 (67.8%) presented within 24 hours of fever onset. ACT was provided to 775 of 1457 (53.2%) patients during RDT weeks and to 1422 of 1473 (96.5%) patients during CD weeks (Odds Ratio (OR) 0.039, 95% CI 0.029–0.053). The CHWs adhered to the RDT results in 1411 of 1457 (96.8%, 95% CI 95.8–97.6) patients. More patients were referred on inclusion day during RDT weeks (10.0%) compared to CD weeks (1.6%). Referral during days 1–7 and perceived non-recovery on days 3 and 7 were also more common after RDT aided diagnosis. However, no fatal or severe malaria occurred among 682 patients in the RDT group who were not treated with ACT, supporting the safety of withholding ACT to RDT negative patients.

**Conclusions/Significance:**

RDTs in the hands of CHWs may safely improve early and well-targeted ACT treatment in malaria patients at community level in Africa.

**Trial registration:**

ClinicalTrials.gov NCT00301015

## Introduction

Malaria remains a leading cause of death among children in sub-Saharan Africa [1] despite reports of reduced burden of disease following wide scale deployment of modern control interventions [2]. Since early diagnosis and prompt effective treatment is essential to prevent fatal malaria, strategies to improve access to treatment at community level have been endorsed by WHO [3], [4]. Early effective antimalarial treatment may also reduce the risk of further transmission. Home-based management of malaria (HMM), where members of the community treat malaria patients, is therefore now being introduced in Africa.

Artemisinin-based combination therapy (ACT) is now generally recommended as first-line treatment for uncomplicated malaria globally [4]. In Africa, ACTs were recently found to be successfully integrated in HMM strategies by community health workers (CHWs) [5], [6]. Malaria diagnosis is still, however, often based on clinical symptoms in many parts of Africa resulting in over-diagnosis and over-treatment. In mainland Tanzania, presumptive malaria treatment is used in febrile children residing in malaria endemic areas, in accordance with the Integrated Management of Childhood Illness (IMCI) strategy, as well as in older patients in health facilities lacking confirmatory diagnostic facilities [7]. Presumptive malaria treatment with ACT is costly, may spur development of drug resistance [8], [9] and prevents other causes of fever from being considered, diagnosed and appropriately treated. A key challenge for improved case management of fever patients in Africa is thus to provide early access to ACT at peripheral and even community level after parasitological confirmation.

RDTs are antigen-based dipstick tests requiring neither a skilled technician nor electricity, which makes them useful even in rural areas [10], [11]. Previous studies have confirmed their high sensitivity and specificity under field conditions [12]–[16]. However, health systems performance remains critical [17] and poor adherence to test results with up to 50% of RDT negative patients treated with ACT in effectiveness studies at health facility level have raised concerns of the usefulness of RDTs [18]–[21]. Conversely, recent studies have reported higher adherence to RDT negative results [22]–[23]. In addition to the adherence issue, there is also debate as to the risk of withholding antimalarial treatment especially in young children who test negative by RDT for malaria [24]–[27].

Limited earlier studies suggest that RDTs can be accurately used by CHWs [10], [28] and recently, Yeboah-Antwi et al reported promising results in Zambia where CHWs, previously trained in curative care, were using RDTs, ACT and antibiotics for management of children with fever [29]. In Tanzania CHWs are normally not trained in curative care.

We studied the impact of RDT-aided management of fever patients as compared with clinical diagnosis (CD) only by CHWs on provision of ACT for uncomplicated malaria at community level.

## Materials and Methods

The protocol for this trial and supporting CONSORT checklist are available as supporting information; see Checklist S1 and Protocol S1.

### Ethics

This study was approved by the Muhimbili University of Health and Allied Sciences (ref MU/RP/AEC/Vol.IV/52) and by the Regional Ethics Committee, Stockholm (2006/2:2). The patients and/or their caretakers signed a written consent form at inclusion, or provided a thumb print after the consent form was read to them.

### Study area and health care

The study was conducted during the peak malaria transmission period (March–August 2006) in Kibaha District, a high-transmission area of Coast Region, Tanzania [30]. Eight villages were initially selected for logistic reasons and for being catered by a common second level health centre (Mlandizi). Five of these eight villages, with a total population of 8625 people, were then selected based on their rural location and limited availability of private health care outside the existing public services.

Each of the five study villages had one village dispensary, representing the first level of public health care. The five dispensaries were open daytime on weekdays and had 2–5 staff members, including one clinical officer, one nurse, and usually a midwife. Sunlight microscopy for malaria diagnosis was available in two of the five dispensaries. By the time of the trial, there was no private health facility, pharmacy or drug vendor in any of the selected villages or within a distance of 10 km. The second level of health care and thus referral facility for the dispensaries in the study area was Mlandizi Health Centre, situated at a distance of 9 to 18 kms from the selected villages. This 24-hour open health centre provided basic laboratory services including malaria microscopy, haemoglobin determination and urine and stool examination as well as inpatient care, but no facilities for surgery, blood transfusions or care for severely ill patients.

### Community health workers

CHWs are an integrated part of the existing health care infrastructure in Tanzania. They are appointed by the community among the village members, but to be eligible, they must have primary education. CHWs are trained in health promotion. In recent years some have received additional training in demographic and health surveillance. However, CHWs do not receive any formal education in health care. Hence, the CHWs had not been involved in any routine curative services prior to the study.

After consultations with village leaders, district authorities and dispensary staff, 22 active CHWs in the five selected villages were asked to participate in the study. All provided informed consent. The study CHWs included 12 men and 10 women, with age ranging from 20 to 50 years. The CHWs were 2–6 per village depending on population size, each CHW being responsible for approximately 300 to 500 people. During the study period each CHW was given a monthly allowance equivalent to 15 USD. The CHWs were mostly farmers working in the vicinity of their homes. They were to be available for any fever patient consultation most of the day and at night.

Before the study, the CHWs received one week specific training on malaria symptoms, performance and interpretation of RDT, prescription of ACT (artemether-lumefantrine), identification of danger signs according to IMCI and indications for referral. The study specific training also included study design, blood sampling and data handling. Instruction booklets for questionnaires and other study procedures were provided to the CHWs. The primary referral level during weekday working hours was the village dispensary or else the Mlandizi Health Centre.

Community sensitization meetings were held prior to the study start when the villagers were informed about the purpose and conduct of the study.

### Study design and outcomes

The intervention consisted of training CHWs in fever case management using CD only or RDT aided diagnosis, and provision of ACT for use in patients diagnosed with malaria. A systematic random sampling data design was implemented. Each of the 22 CHWs was assigned a unique number which was noted on a lottery ticket. Eleven tickets were then picked blindly by one researcher (AJ) from a box after mixing. The selected eleven CHWs were allocated to start using RDT the first study week and thereafter alternating with CD weekly. The remaining eleven CHWs were allocated to use CD the first week. This cross over design at CHW level was applied to account for seasonal and geographic differences in malaria transmission and variations in CHWs' individual performance.

The primary outcome measure was proportion of patients provided ACT during RDT and CD weeks. Secondary outcomes included proportion of patients presenting within 24 hours of fever onset, referral rates up to day 7, health outcome by day 3 and 7, compliance to treatment and mortality up to day 28.

### Patient inclusion, diagnosis and treatment

The sample unit was a patient with reported fever. The inclusion criteria were history of fever in the preceding 24 hours in patients above 3 months of age and informed consent from the patient and/or parent/guardian. Exclusion criteria were pregnancy, symptoms suggestive of severe disease and prior study inclusion within the previous 28 days.

CHWs were to provide ACT based either on symptoms suggestive of uncomplicated malaria during CD weeks and symptoms together with an RDT positive result during RDT weeks. Thick blood smears were collected through finger pricks from all enrolled patients. All blood smears were sent to a field research laboratory established at Mlandizi Health Centre for Giemsa staining and microscopical investigation. The microscopy results were thus not available to guide treatment. Artemether-lumefantrine (Coartem®, Novartis, Basel, Switzerland) was to be administered according to body weight/age groups in six doses over three days as recommended by the manufacturer. Pre-packaged doses with written information and illustrations for four different weight/age groups were provided to the CHWs to ease drug dispensing. The first dose of ACT was to be given under CHW supervision. The patients/guardians were recommended to preferably administer the remaining drug doses together with fatty food. Paracetamol was provided to all fever patients. CHWs were instructed to refer patients that they considered in need of other or additional treatment to the village dispensary or if necessary, to Mlandizi Health Centre.

### Data collection and follow-up

Study questionnaires for data collection had been developed, translated into Swahili and piloted and revised during the training of CHWs. Field supervision for technical support, refill of supplies, and collection of forms and blood smears of the CHWs were done regularly throughout the study. Patients were asked to return to the CHW routinely on days 3 and 7 and any day if they did not improve or if symptoms deteriorated and finally also if they got fever again within 28 days after enrolment. The CHW collected information on patient's subjective general health using four categories (“fully recovered”, “better but still sick”, “same”, “worse”) and also possible side effects of ACT and intake of any additional treatment, e.g. antibiotics. The CHW also collected a blood smear at the routine check up day 7 and anytime a patient returned with fever. For the day 3 follow-up, the patients were asked to bring the ACT blister package to assess treatment compliance. Patients who did not return for the scheduled day 7 visit were actively followed-up at home by the CHW to collect health status information from the patient, guardian, relative or neighbour.

### RDT and microscopic examination

RDTs were used according to manufacturer's instructions (Paracheck Pf, Orchid Biomedical Systems, Goa, India). CHWs were asked to save the positive RDTs for cross checking of the result by the study team within a week from test performance.

Thick blood smears collected by the CHWs were sent to Mlandizi where one study laboratory assistant performed Giemsa-staining and microscopic examination in light microscopy (Olympus) with oil immersion objective (magnification×1000). Asexual parasites were counted against 200 white blood cells and parasite densities calculated assuming a white blood cell count of 8000/µl blood. A blood smear was defined as negative if no parasites were detected after ten minutes' reading. Quality control was performed by two experienced technicians blinded to the initial results at the Department of Parasitology, Muhimbili University of Health and Allied Sciences (2^nd^ reader) and for 10% of the blood smears at the Malaria Research Unit, Karolinska Institutet (3^rd^ reader). The result from the second reading was decisive whenever discordant with field laboratory result.

### Statistics and data analysis

Sample size calculation was done for independent data aiming at 80% power and 5% significance level using Stata 10.0 (StataCorp LP, US). To detect a reduction in ACT prescriptions with RDT aided diagnosis from 80% to 70%, each age group (below 5 years of age, 5–14 years and 15 years and above) should contain at least 314 patients. Given the study design, clustering at CHW level (22 CHWs) was taken into account. For a sample of 480 patients per age group, the maximum intra-cluster coefficient (ICC) values could be 0.025, still reaching 80% power [30]. The planned study period was 16 weeks but patient recruitment was slower than expected and enrolment was therefore extended to 20 weeks targeting 360 children below 5 years of age, 360 aged 5–14 years and 720 aged 15 years and above for RDT and CD weeks, respectively, with an estimated 1∶1∶2 attendance ratio. A sample of 360 patients per age group would reach a power of 80% for ICC values up to almost 0.01.

Data were entered in EpiData 3.1 (http://www.epidata.dk), cleaned and analysed using EpiInfo 6.04d (CDC/WHO) and Stata 10.0 (StataCorp LP, USAll frequencies, proportions and odds ratios (ORs) presented were calculated with 95% confidence intervals (CIs) and corresponding p-values. The ORs and their standard errors were adjusted for clustering on CHW level using mixed effect model. For the main outcome variables data are presented for the three age groups. Significances are stated at the 5% level.

## Results

**Figure 1:**
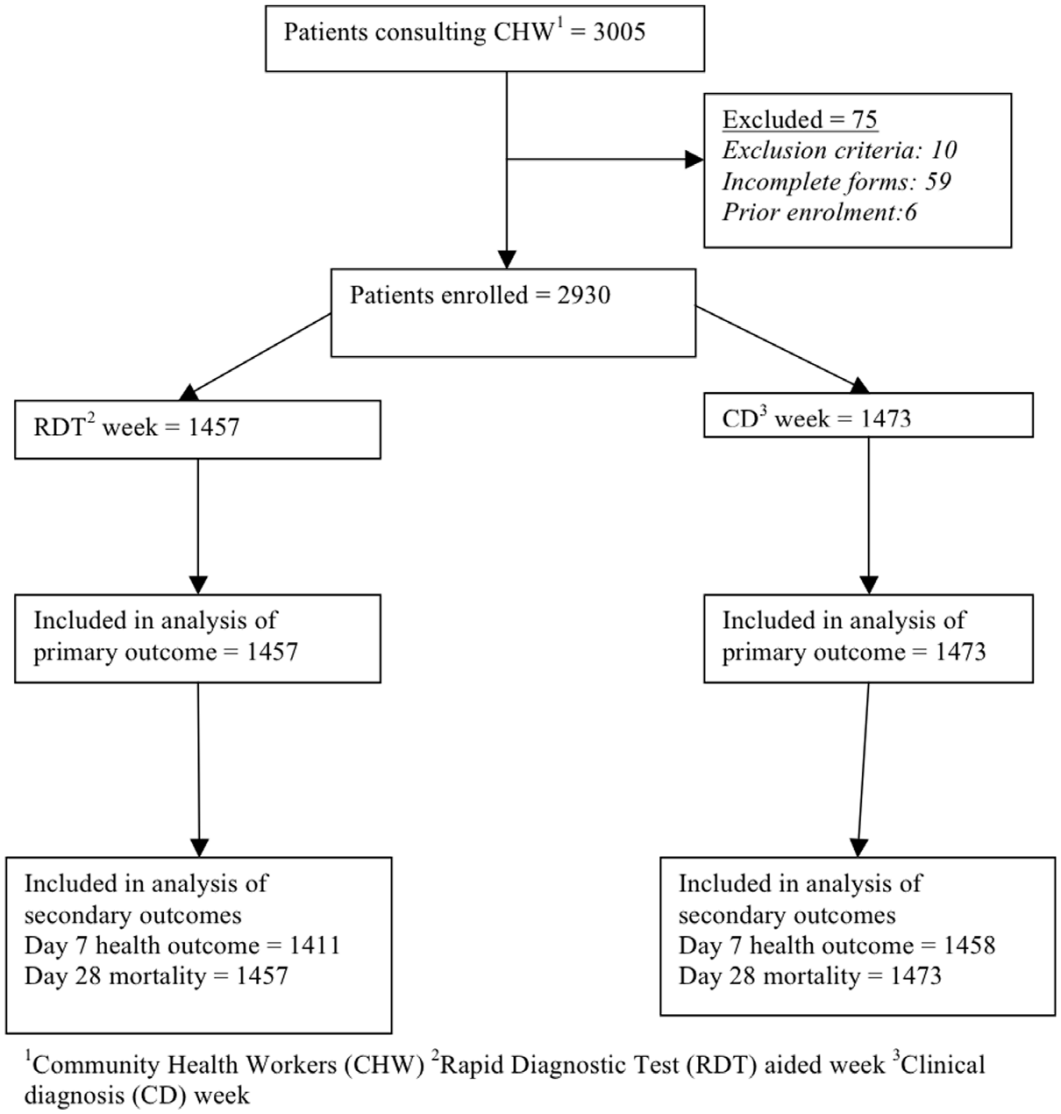
Flow of patients through the study.

During the 5-month study period 3005 patients with fever, of whom 75 were excluded, consulted the 22 CHWs (Figure 1). The remaining 2930 patients, including 760 children below 5 five years of age, were thus included in the analysis. Some 153 and 7 individuals were enrolled twice and three times, respectively, with fever episodes occurring more than 28 days apart. Each CHW enrolled a mean of 133 patients (range 60–265). One CHW was withdrawn from the study after 18 weeks due to absenteeism.

There was a balanced enrolment during the two different intervention weeks, 1457 (49.7%) patients during RDT and 1473 (50.3%) during CD weeks. The enrolment was also found to be balanced in terms of sex, age groups and duration of fever which indicates that health seeking behaviour among patients was not affected by the respective diagnostic intervention. Patient baseline characteristics are presented in Table 1. A total of 1988 (67.8%) patients including 486 (63.9%) children below 5 years of age presented within 24 hours after onset of fever. Some 218 (7.4%) patients presented at night.

**Table 1:** Baseline characteristics of patients enrolled during Rapid Diagnostic Test (RDT) and Clinical Diagnosis weeks.

Characteristics	RDTN (%)	Clinical DiagnosisN (%)
Total number	1457	1473
Females	773 (53.1)	796 (54.0)
Age groups (years)		
<5	368 (25.3)	392 (26.6)
5–14	380 (26.1)	365 (24.8)
≥15	709 (48.6)	716 (44.6)
Duration of fever (hours)		
<24	981 (67.3)	1007 (68.4)
24–48	347 (23.8)	332 (22.5)
>48	129 (8.9)	134 (9.1)

During RDT weeks, 733 of 1457 (50.3%) patients were RDT positive (Table 2), whereas 1416 of 1473 (96.1%) were clinically classified as malaria during CD weeks. 732 out of 733 RDTs interpreted as positive by CHWs were cross-checked by the research team and only 2 (0.3%) had no activity band. The microscopical reading in Mlandizi (first reader) detected parasites in 643 of 2841 (22.6%) blood slides. The blood slide positivity rates in the three age groups were 37.8%, 28.0% and 12.0%, respectively. The sensitivity and specificity of RDTs compared to microscopy results were 85.3% and 59.8%, respectively. Staining quality was partly poor with auto-fixation of the thick films. The field and central laboratory level microscopy results agreement was 85% (kappa coefficient = 0.59). The sensitivity and specificity of the initial field blood slide reading versus central laboratory result was 69% and 90%, respectively.

**Table 2:** Proportions of patients prescribed artemisinin-based combination therapy (ACT) in Rapid Diagnostic Test (RDT) and Clinical Diagnosis arms by age group.

	RDT					Clinical Diagnosis (1)		
Age group(years)	Total	RDT positive		ACT treated		Total	ACT treated	
		n	% (95% CI)	n	% (95% CI)		n	% (95% CI)
<5	368	205	55.7 (47.1–64.3)	215	58.4 (49.6–67.2)	392	381	97.2 (94.9–99.5)
5–14	380	258	67.9 (60.4–75.3)	268	70.5 (64.1–76.9)	365	350	95.9 (91.5–100)
≥15	709	270	38.1 (33.3–42.8)	292	41.2 (35.5–46.9)	716	691	96.5 (92.4–100)
All patients	1457	733	50.3 (45.7–54.9)	775	53.2 (47.7–58.6)	1473	1422	96.5 (93.5–99.5)

(1) A total of 1416 were classified as probable malaria but an additional 6 (total 1422) were ACT treated.

ACT was provided to 775 of 1457 (53.2%) patients during RDT weeks and to 1422 of 1473 (96.5%) patients during CD weeks (OR 0.039, 95% CI 0.029–0.053) (Table 2). The overall relative reduction in provision of ACT during RDT weeks was thus 44.9%, and most prominent in patients 15 years and above (57.3%). ACT was provided to 44 of 724 (6.1%) patients with RDT negative results, whereas two patients were not treated despite being RDT positive. Overall CHW adherence to RDT results was thus recorded in 1411 of 1457 (96.8%, 95% CI 95.8–97.6) patients. During CD weeks, 6 of 57 (10.5%) patients received ACT despite being clinically classified as non-malaria.

Referral occurred more often in the RDT group compared to the CD group (Table 3). Combining day 0 and day 1 to 7, 250 of 1457 (17.2%) patients were referred in the RDT group compared to 73 of 1473 (5.0%) in the CD group (OR 4.3, 95% CI 3.2–5.7). Among the 146 patients referred on day 0 in the RDT group, only 12 (8.2%) received ACT before referral. Similarly, in the CD group, referral on day 0 was relatively more common among patients who did not receive ACT. Main reasons for referral (80%) were “fever but not malaria” (RDT negative), abdominal pain/diarrhoea/vomiting, cough/chest pain and difficult breathing. Other reasons included ear/eye problems, skin diseases, worms, body pains, tonsillitis and flue.

**Table 3:** Proportions of patients referred to dispensaries following Rapid Diagnostic Test (RDT) and Clinical Diagnosis.

	RDT			Clinical Diagnosis			OR* (95% CI)	P-value
	Total	Referred	% (95% CI)	Total	Referred	% (95% CI)		
Referral Day 0								
<5 years	368	41	11.1 (7.9–14.4)	392	14	3.6 (1.7–5.4)	3.85 (1.97–7.53)	0.000
5 –14 years	380	28	7.4 (4.7–10.0)	365	4	1.1 (0.0–2.2)	7.35 (2.50–21.61)	0.000
≥15 years	709	77	10.9 (8.5–13.2)	716	6	0.8 (0.1–1.5)	33.53 (13.19–85.18)	0.000
Not treated	682	134	19.6 (16.6–22.6)	51	8	15.7 (5.6–25.8)	0.76 (0.29–1.98)	0.578
Treated	775	12	1.5 (0.6–2.4)	1422	16	1.1 (0.5–1.7)	1.51 (0.69–3.27)	0.297
All patients	1457	146	10.0 (8.4–11.6)	1473	24	1.6 (0.9–2.3)	9.16 (5.77–14.54)	0.000
Referral Day 1–7								
<5 years	368	35	9.5 (6.5–12.5)	392	14	3.6 (1.7–5.4)	2.84 (1.47–5.46)	0.002
5–14 years	380	12	3.2 (1.3–4.9)	365	4	1.1 (0.0–2.2)	2.92 (0.90–9.44)	0.073
≥15 years	709	57	8.0 (6.0–10.0)	716	31	4.3 (2.8–5.8)	1.53 (0.94–2.47)	0.082
Not treated	682	78	11.4 (9.0–13.8)	51	3	5.9 (0.6–12.4)	1.92 (0.52–7.11)	0.327
Treated	775	26	3.4 (2.0–4.6)	1422	46	3.2 (2.3–4.2)	0.92 (0.55–1.52)	0.747
All patients	1457	104	7.1 (5.8–8.5)	1473	49	3.3 (2.4–4.2)	1.95 (1.36–2.80)	0.000

*ORs are adjusted for clustering on CHW level, see Statistics and data analysis-section.

Four patients died during the trial. Three children below 5 years of age died within three days and one adult within seven days after enrolment. Malaria diagnosis was confirmed by microscopy in two deceased children (32,800 and 54,000 parasites/µl, respectively). Both had initially been diagnosed with malaria (after RDT and CD, respectively), prescribed ACT by the CHW and referred for further management. The third child was both RDT and blood smear negative and was not treated with ACT, but referred directly to the dispensary due to breathing problems. Similarly, the deceased adult was also RDT and blood smear negative, and referred for management of stomach pain on day of enrolment. She reattended on day 3 with unchanged condition and was again referred to the dispensary by the CHW. Apart from the two deceased children with confirmed malaria infection no other patients developed severe malaria.

Health outcome by day 3 and 7 in the RDT and CD group is presented in Table 4. Information about health outcome by day 7 was obtained from 2869 of 2930 (97.9%) patients. The remaining 61 patients had left the study area at the time of follow-up and were thus lost to follow-up.

**Table 4:** Reported health outcome by day 3 and 7, respectively, in patients enrolled during Rapid Diagnostic Test (RDT) and Clinical Diagnosis weeks.

	RDT			Clinical Diagnosis			OR* (95% CI)	P–value
	Total	n	%	Total	n	%		
Full recovery Day 3								
<5 years	336	274	81.5	382	342	89.5	0.55 (0.34–0.87)	0.010
5–14 years	347	312	89.9	360	344	95.6	0.45 (0.23–0.89)	0.021
≥15 years	652	529	81.1	701	638	91.0	0.50 (0.34–0.71)	0.000
Treated	760	687	90.4	1399	1295	92.6	1.03 (0.73–1.44)	0.872
Not treated	575	428	74.4	44	29	65.9	2.47 (1.09–5.58)	0.030
All patients	1335	1115	83.5	1443	1324	91.8	0.52 (0.40–0.67)	0.000
Full recovery Day 7								
<5 years	360	342	95.0	386	372	96.4	0.75 (0.36–1.53)	0.431
5–14 years	370	361	97.6	364	362	99.5	0.22 (0.04–1.03)	0.055
≥15 years	681	614	90.2	708	684	96.6	0.35 (0.15–0.57)	0.000
Treated	764	743	97.3	1408	1371	97.4	1.11 (0.65–1.88)	0.704
Not treated	647	574	88.7	50	47	94.0	0.53 (0.15–1.90)	0.333
All patients	1411	1317	93.3	1458	1418	97.3	0.45 (0.30–0.65)	0.000

*ORs are adjusted for clustering on CHW level, see Statistics and data analysis-section.

Full recovery was more commonly reported in the CD group (97.3%) compared to the RDT group (93.3%). Among the non-recovered patients in the RDT group, 32 (27 RDT negative on day 0) reported unchanged or worse condition on day 3, and 10 on day 7. The corresponding numbers in the CD group were 8 and 4, respectively. Among the 14 patients with unchanged or worse condition on day 7, only one (CD group) had a positive blood slide on day 7 (5,400 parasites/µl). Among 120 patients reporting improvement but not complete recovery on day 7, three were positive by microscopy, one (30,320 parasites/µl) in the RDT group and two (35,600 and 45,200 parasites/µl) in the CD group. Two of the four parasite positive patients on day 7 had been prescribed ACT on day 0.

ACT treated patients more often reported full recovery than ACT untreated, i.e. 97.3% versus 89.1% (OR 4.4, 95% CI 3.1–6.3). However, the only two non-ACT treated patients despite positive RDT result reported full clinical recovery by day 7 and severe malaria did not develop in any of the overall 682 patients in the RDT group who were not treated with ACT.

Withholding ACT in children with fever represents a special concern. Among the 153 children below 5 years of age in the RDT group, who were not treated with ACT, 26 were referred on day 0 and 3 upon reattendance on days 1–7. All blood slides (n = 40) collected on day 7 routine follow-up were negative by microscopy. This included blood slides from the 3 children who reported they were not fully recovered.

During the 8–28 day passive follow-up a total of 16 patients (5 RDT and 11 CD), reattended the CHWs due to fever, of whom seven were below 5 years of age and three had positive microscopy (3,000, 5,840 and 123,520 parasites/µl) between day 23 and 28. All three were from CD group and had received ACT at enrolment.

Full ACT compliance, i.e. intake of whole regimen according to dose schedule was reported by 2123 of 2156 (98.5%) ACT treated patients who returned for day 3 follow-up. Reported compliance was higher during CD-weeks (99.3%) than during RDT-weeks (97.4%), (OR 3.3, 95% CI 1.5–7.7). Altogether 2115 patients (96.3%) returned their ACT blister packages and 99.2% of these were empty.

Adverse events interpreted as drug related were reported by 24 patients (1.1%). No drug related severe adverse event was reported. The most common complaints were nausea, weakness, headache and diarrhoea. Incomplete compliance to whole ACT regimen was reported by 10 of these 24 patients compared to only 20 of 2131 patients not reporting adverse event (OR 143, 95% CI 43–500).

## Discussion

This study demonstrates that the use of RDTs by CHWs can be safe and effective for targeting ACTs to patients with uncomplicated malaria in sub-Saharan Africa [29]. RDT use significantly reduced prescription of ACT by 45% as compared with CD, with the most striking reduction of treatment for patients above 15 years (57%). Although appropriate use of ACT is especially important in children, reduced over-use of ACT among adults will be more drug-cost saving besides preventing unnecessary drug exposure. The cross-over design of the study may potentially influence attendance to either CD-week (higher chance for ACT) or RDT-week (benefit of the RDT). However, neither preference was observed since very similar attendances were found in CD- and RDT-weeks, respectively (Table 1).

The reduced provision of ACT after RDT use was achieved following high adherence to RDT negative results. This may be related to the specific CHW training and pre-study sensitization of the community. Previous findings of poor adherence to negative results at health facility level have been attributed to poor quality of test and fear of false negative results [10], [19] even after a three-day training [20]. However, with a relatively short training high adherence can also be achieved at primary health care level [22], [23]. It may also be that CHWs are more likely to comply with simple clinical guidelines than other health care workers and that they are relatively well suitable to influence patient compliance to treatment [29], [31]. The reported patient compliance to the treatment (>95%) suggests that the complex regimen of artemether-lumefantrine (6 doses over 3 days), as compared to the previous single dose treatment of sulphadoxine-pyrimethamine (SP), did not discourage patients from completing the treatment. However, the study design with active follow-up and collection of blister packages may also have influenced compliance to the full-dose regimen. High community acceptability of ACT when prescribed at community level has been described in two previous studies conducted in sub-Saharan Africa but they did not address compliance [5], [32].

The health impact of RDT and targeted ACT will depend on the safety of withholding ACTs and the benefit of additional non-malaria treatment to RDT negative patients. Interestingly, no fatal or severe malaria was reported among the patients not treated with ACT. The two malaria patients with fatal outcome had both been prescribed ACT and most importantly all 4 patients with fatal outcome had been identified by the CHWs as potentially severely ill and consequently referred to the village dispensary. Considering that our study was conducted in a rural setting, where any news of severe disease and death is likely to reach the CHW instantly, we assume that the risk of any death or severe malaria manifestation other than the reported is limited.

The potential benefits from increased attention to non-malarial illnesses in RDT negative patients could not be documented in our study. However, based on the RDT results, at least 50% of patients during the CD weeks did not have malaria as cause of fever and were potentially therefore incorrectly treated with ACT only. Approximately 20% of RDT-negative patients were referred compared to about 2% among RDT positive patients or patients from the CD group. This suggests that the CHWs were well aware that negative RDT results could indicate presence of diseases other than malaria, which may need non-malarial treatment. Despite this, the self-reported full recovery rate by day 7 was higher during CD than RDT weeks. This may partly reflect satisfaction of having been ACT treated, but it may also reflect remaining symptoms in non-treated patients with low density parasitemia undetected by RDT. The high recovery rate in the CD group also suggests that most non-malaria fevers represent self limiting diseases, which do not require other treatment such as antibiotics.

In our study, over 60% of the patients presented at the CHWs within 24 hours of fever onset. In a recent report from Kenya only 23% of febrile children received ACT at health facility level within 48 hours [33], which is far from the target of treating 80% of malaria episodes within the first 24 hours by 2010 [34]. In the recent Tanzania Demographic and Health Survey 2010 based on a national sample of 10300 households, 21% of children under five were reported to have had fever in the two weeks preceding the survey. Of these, 58% were given any anti-malarial treatment, but just 28% took ACT within 48 hours [35].

Our results indicate that the CHW may represent an important provider of early fever case management and thus access to ACT in rural settings in Africa where many people live at considerable distance from health care facilities [29], [36]. In two previous studies improved child survival has generally been observed when curative services at community level have been provided by nurses [37], but not by community volunteers [38]. A few smaller studies on community or home-based management of malaria have shown varying results on health outcomes [39].

The overall RDT positivity rate observed was higher than the blood slide positivity rate. This may reflect false positivity (low specificity) or higher sensitivity with RDT. False positive results with HRP2 based RDTs, such as the one used in our trial, may be due to persistent antigenemia in individuals recently treated for malaria [40]–[42] and low specificities with HRP2 based RDTs (about 70%) compared to microscopy have also been documented in previous studies when used in primary health care settings [41], [43], [44]. However, the higher positivity rate of RDTs retrieved in our study compared to microscopy is probably also influenced by the partly poor quality of blood slides. The validity of microscopy in peripheral health facilities has previously been questioned because of technical problems [45] and RDT results may thus turn out to be more accurate than microscopy, especially when the blood tests are performed at community level by CHWs.

### Conclusion

The role of CHWs in providing curative care is a matter of debate. This study suggests that malaria RDTs in the hands of CHWs provide an opportunity to improve access to early, well targeted ACT treatment at community level as well as better opportunity for non-malaria treatment, such as antibiotics for pneumonia, at referral level. This is expected to reduce severe disease and death in malaria and other diseases and also to reduce the risk of development of ACT resistance. However, the effect and impact of RDT including its cost effectiveness [15], [46] will depend on the local malaria endemicity and the accuracy of the selected type of RDT in that local context. More studies are now also required to improve management of non-malaria ( = RDT negative) febrile patients including an optimal integration of CHWs into the formal health care system.

## Supporting Information

Checklist S1 CONSORT checklist
(DOC)

Protocol S1 Trial Protocol
(PDF)
