# Supplementary material for: Malaria Rapid Testing by Community Health Workers Is Effective and Safe for Targeting Malaria Treatment: Randomised Cross-Over Trial in Tanzania
Source: PLoS One. 2011 Jul 5;6(7):e19753. doi: 10.1371/journal.pone.0019753 (PMC3130036; doi:10.1371/journal.pone.0019753)
Supplement: Protocol S1 — Trial Protocol (PDF) [file pone.0019753.s002.pdf]

Title:	**Rapid Diagnostic Testing and Artemisinin - Based Combination Therapy for Uncomplicated Malaria by Community Health Workers**
Short Acronym:	RapAct
Long Acronym:	RDT and ACT for malaria by CHWs
Updated on:	28 February 2006 (Final +1)
Design:	Multicentre comparative crossover validation study
Study sites:	Villages in Kibaha District
Study Director:	Anders Björkman, KI
Principal Investigators:	Annika Janson, KI<br>Zul Premji, MUCHS
Co-Investigator(s):	Marycelina Mubi, MUCHS (RDT Study)<br>Gloria Maganga, MUCHS (RDT study)<br>Billy Ngasala, MUCHS (Coartem Study)
Technical Advisors:<br>(Protocol Development)	Karin Källander, KI (RDT study)<br>Andreas Mårtensson, KI (RDT + Coartem study)
Technical Collaborators:<br>(Laboratory Part)	Yngve Bergqvist, Falun, Sweden (Coartem study)<br>Pedro Gil, KI (Coartem study)<br>Andreas Mårtensson, KI (Coartem study)<br>Christin Sisowath, KI (Coartem study)
Data Management and<br>Study Monitoring:	Karin Källander, KI<br>Max Petzold, Gothenburg<br>Marian Warsame, KI
Steering Committee:	Zul Premji, MUCHS<br>Amos Massele, MUCHS<br>Göran Tomson, KI<br>Lars Gustavsson, KI
Sponsor:	MUCHS-KI QC PROJECT (Sida/SAREC)

Table of Contents

No.	Titles:	Page
1.	Background and Relevance	
1.1.	Malaria Treatment and ACT	
1.2	Malaria diagnosis and RDT	
1.3	Community based diagnosis and treatment	
1.4	Local background	
1.5	General rationale	
2.	Hypotheses	
3.	Objectives	
3.1	General aim	
3.2	Study objective	
3.3	Specific objectives	
4.	Methods	
4.1	General study design	
4.2	Study area and sample size	
4.3	Sample unit	
4.4	Specific design	
4.5	Inclusion criteria	
4.6	Exclusion criteria	
4.7	Diagnosis and treatment	
4.8	Analysis	
4.9	Outcomes	
4.10	Study Power/sample size estimation	
4.11	Study products	
4.12	Study sites	
4.13	Enrolment of patients	
4.14	Follow-up	
4.15	Randomisation	
4.16	Concomitant drug therapy	
4.17	Collection of blood samples and performance of laboratory tests	
4.18	Follow-up (See flow chart)	

4.19	An Integrated Referral System
4.20	CHW follow-up questionnaire/focus group interviews
4.21	Monitoring/supervision
4.22	Study registration
4.23	Data Management and evaluation
4.24	Data dissemination and publication
4.25	Ethical considerations
4.26	Ensuring an effective and sustainable study drug supply system
5.	Capacity strengthening
5.1	Community level
5.2	Field Research Team
5.3	Research and Training Programme
5.4	Laboratory technology transfer
6.	Study Components and Time Plan
6.1	June 15th – December 15th *(Ongoing activities at different levels of implementation)*
6.2	Preparations in the field: Ongoing activity started in August 2005
6.3	Teaching period (*20th - 24th February 2006*)
6.4	Intervention phase (March to June 2006)
6.5	PCR and drug analyses (For details – see Coartem study proposal)
6.6	Data analysis (To be determined) will be performed mainly in Stockholm.
6.7	Inputs of Technical Advisors and Collaborators
6.8	Field Research Team (see Appendix 1)
7.	Time plan
8.	Budget
8.1	RapAct budget in Tanzania (Appendix 2)
8.1.1.	Development of HQ in Mlandizi
8.1.2	Workshop 16-20, January 2006
8.1.3	Master budget
8.2	RapAct budget in Sweden
9.	References

Appendices:

Appendix 1	Health facilities in Kibaha
Appendix 2	Logistic plan
Appendix 3	CRF and Consent Forms
Appendix 4	RapAct budget in Sweden
Appendix 5	Master budget for RapAct in Tanzania
Appendix 6	Ethical clearances
Appendix 7	Research Team

Study Proposal:

RapAct

Rapid Diagnostic Testing and Artemisinin – Based Combination Therapy for Uncomplicated Malaria by Community Health Workers

1. Background and Relevance

1.1 Malaria Treatment and ACT

In the Roll Back Malaria (RBM) strategy and the Abuja Malaria Summit one of the six main targets is that 60 % of those suffering from malaria should have access to and be able to use correct, affordable and appropriate treatment within 24 hours of the onset of symptoms (The Africa Malaria Report, 2003).

Due to resistance of *Plasmodium falciparum* malaria to common antimalarial drugs and its health implications, most African countries are switching to artemisinin-based combination therapy (ACT) as first line treatment policy (Björkman & Bhattarai, 2005). Currently, the resistance to the first-line drug in Tanzania since 2001, sulfadoxine-pyrimethamine (SP), is developing rapidly, with high resistance pockets reaching a rate of 15 % in many areas of the country, and up to 34% in Muheza area (Hastings et al., 2002).

In Zanzibar, ACT has been implemented since 2004, and the mainland of Tanzania has decided to switch from SP to ACT in 2006. Zanzibar is using artesunate+amodiaquine as the first line treatment and Coartem®, a fixed dose combination of artemether-lumefantrine, as second line option. On the mainland Coartem will be first line treatment with quinine monotherapy as second line.

The necessity to introduce more expensive ACTs is making diagnostics of true malaria infection more important than before. The concept of home-based management, where fever alone is the indication to treat, is therefore challenged due to its built-in risk of over-treatment (O'Dempsey et al., 1993; Mkandala et al., 2000; Nsimba et al., 2003). Over-treatment increases costs and the risk of development of resistance towards the new drugs. On the other hand, a delay in diagnosis and treatment due to a lower accessibility to the correct drugs would greatly harm the possibility of fulfilling the Abuja target stated above. Evidence on the

use of ACT in community management is lacking but highly needed. For example, experience from Uganda is based on the use of chloroquine (Kallander et al., 2004).

The cost of ACT ranges from 0.9 USD for treating a child to 2.4 USD for treating an adult (Piola et al., 2005). This is making ACT 10-20 times more expensive than the current regime in Tanzania, which is SP. The price of rapid diagnostic test (RDT) (0.5-1 USD) is motivated if the diagnostic accuracy reduces the use of ACT without affecting health outcome. The cost effectiveness of RDT will depend on the prevalence of disease in a specific area.

1.2 Malaria diagnosis and RDT

Malaria is the leading cause of morbidity in Tanzania, accounting up to 30% of outpatient attendances and admissions at health facilities. In most of the peripheral health facilities, equipment and trained personnel to perform laboratory diagnosis of malaria are not available, and diagnosis is based on clinical criteria, mainly presence or history of fever. Unfortunately, clinical criteria for a clinical episode of malaria are quite non-specific, although "erratic fever" has been shown to be highly predictive/specific for malaria associated fever (Rooth & Björkman, 1992). The Swahili wording for this is "homa ya vipindi". To our knowledge, the value of integrating this term in clinical algorithms has not yet been tested in alternative studies.

The RDT provides a new, simple and accurate way of determining malaria infection. Such a test in the form of dipsticks or plates are based on detection of parasite antigen in blood, and do not require the skills of a microscopist nor investments like microscope and electricity.

Evaluations of RDTs have been carried out in different settings and have shown that there is wide variability within and between products, which calls for improvement. However, most of the tests were found to be sensitive and specific, with rates of 94-100% and 89-97% respectively (Singh, Saxena & Sharma, 2002; Singh & Saxena, 2005), taking a short time to produce results (5-15 minutes), and can be performed by personnel with minimal training (Arora et al., 2003, Bell et al., 2001, Aslan et al., 2001). They indicate presence or absence of parasites, and whether the infecting species is *Plasmodium falciparum* or non-falciparum. The ability to detect low levels of parasitemia may, however, be considered a disadvantage in endemic areas, where many people carry parasites without being sick. Patients with fever and low density parasitemia may thus be mistaken as malaria patients.

Another issue is that some RDT tests, especially those based on detection of Histidine-rich protein 2 (HRP2), remain persistently positive up to three weeks after clearance of parasitemia, which has been attributed to persistence of antigens and presence of gametocytes (Arora et al., 2003; Singh & Shukla, 2002; Tjitra et al., 2001). Such tests should not be used to monitor treatment outcome.

The tests are relatively expensive, at 0.5-1 USD per test (WHO, 2000; Moody, 2002). For this reason, they were not considered cost effective for widespread use during the era of chloroquine and SP (Wongsrichanalai, 2001). However, they may well be worthwhile when treatment policies change to the more expensive ACT.

1.3 Community based diagnosis and treatment

With the new approach advocated by WHO/RBM to control morbidity and mortality, i.e. making antimalarial treatment available as close to the home as possible, more people without clinical training and hence without the ability to differentiate malaria from other fevers will be involved in providing treatment. This calls for the use of simple and objective criteria in making a diagnosis of malaria.

The most likely persons to be used in Tanzania in extending the availability of antimalarials to the households are Community Health Workers (CHWs). These are community members, usually two in each village (male and female) who have been identified by village health committees to deal with health issues in their communities. They are involved in mobilizing communities and assist health workers in health facilities during Mother and Child Health care (MCH) clinics, vaccinations, and follow up of patients in their communities. In some cases, education seminars are organized for CHW on different health aspects. CHWs are not employed and hence not paid a salary. However, they are supposed to be given a token by their villages.

1.4 Local background

The study will be carried out in Kibaha district, one among five districts of the Coast Region. The district is situated 40-80km west of Dar es Salaam. It is a mainly rural area with about 130,000 inhabitants (12% under the age of five) (National Census of 2002). Kibaha has two administrative councils, and 21 public health facilities (one hospital, one health centre and 19

dispensaries). There are three additional military dispensaries, three dispensaries connected to schools and a public cooperation, as well as three private dispensaries.

The district is in the equatorial rain forest geographical and climatic region, with heavy rainfalls (from 1000-2000 mm per annum) and temperatures from 24ºC to 30ºC. Abundant fresh water, collecting the year round, in creeks, swamps, pools and rice fields ensure the breeding of large numbers of *Anopheles gambiae* and *funestus* mosquitoes, which are the main vectors of malaria. Malaria transmission in the district occurs throughout the year (holoendemic pattern of malaria transmission), with some accentuation during the rainy season (May-July) and to a lesser extent December - January.

Among the strategies for controlling malaria adapted in the Coast Region is treatment of malaria patients with effective antimalarial drugs and the use of insecticide treated nets (ITN). The bed net voucher system is in place, where pregnant women can buy ITNs at subsidized rates to protect themselves and their children.

The MUCHS/KI Malaria group has been involved in malaria research in both Bagamoyo and Kibaha, two of the districts in the Coast region. The research group has completed an intervention to improve malaria diagnosis at health facilities. Kibaha was chosen for this phase of malaria management at community level for several reasons. This district has a well developed structure of CHWs that have been trained in community integrated management of childhood disease (c-IMCI), although they have not previously been involved in curative care. It also has a well planned organisation to support CHW from each dispensary and from a special unit at the district level. Kibaha district can be considered as a model for involvement of community health workers in preventive health care and monitoring of good health practices in their villages.

1.5 General rationale

a) There is increasing evidence that correct diagnosis and prompt and appropriate treatment with effective drugs is a highly cost-effective malaria control strategy. Trained CHWs have been suggested as a tentative delivery channel for provision of antimalarial drugs in order to improve prompt treatment.

b) Previous studies on community based management of malaria have used chloroquine but none have explored the possibility of providing ACT therapy through CHWs.

c) RDTs provide a simple and accurate way of determining malaria infection. Studies have found different RDTs to be sensitive and specific (Singh, Saxena & Sharma, 2002; Singh & Saxena, 2005), taking a shorter time to produce results (5-15 minutes) than microscopy, and they can be performed by personnel with minimal training (Arora et al., 2003; Bell et al., 2001; Aslan et al., 2001). We will evaluate the feasibility of using trained CHWs aided by RDT to provide Coartem for malaria cases in the community.

This study represents an expressed priority by the Ministry of Health (MOH) in Tanzania and will provide results within a relatively short time-span, preceding the change of antimalarial policy in Tanzania and hence likely to have an important effect in this transformation.

Our design is addressing a controversial matter, as stated by the Board on Global Health (BGH) in Saving Lives, Buying Time: Economics of Malaria Drugs in an Age of Resistance 2004).
"However, in high prevalence areas of sub-Saharan Africa, universal treatment of clinically suspected cases with ACTs is the safest near-term strategy, given the desperate need for rapid, in- or near-home treatment in order to save lives. Eventually, combining RDTs and ACTs in areas of Africa of low and medium malaria prevalence could reduce the number of illnesses misdiagnosed as malaria and yield major savings in terms of drug cost and delayed onset of drug resistance. In the meantime, following the introduction of ACTs in Africa, pilot programs and operational research combining ACTs and RDTs in a variety of epidemiologic settings and at-risk populations should be encouraged."

2. Hypotheses

Hypothesis 1: RDT used by CHWs for the identification of non-severe clinical malaria will reduce use of Coartem by 30 % without affecting health outcome.

Hypothesis 2: The effectiveness/parasitological cure of Coartem for the treatment of uncomplicated malaria at community level is above 90 % as assessed in a 42 day follow up in children <5 years old.

Hypothesis 3: The use of RDT and Coartem is well perceived by the community and will enhance health seeking behaviour.

3. Objectives

3.1 General aim: To explore malaria case management with Coartem with or without RDTs by trained CHWs at community level.

3.2 Study objective

To test the efficiency and effectiveness of using RDT's and the effectiveness of Coartem by CHWs for management of uncomplicated malaria at community level and to study the attitudes of these procedures by the community.

3.3 Specific objectives

1. To determine the proportion of children and adults who receive malaria treatment (ACT) from CHWs within 24 hours of onset of symptoms vs. later.

2. To determine the ability of CHWs to correctly assess the presence or absence of malaria using RDT's.

3. To explore reasons for potentially treating RDT negative patients with Coartem.

4. To assess the predictive value of erratic fever ("homa ya vipindi") as a clinical tool to differentiate malaria from other causes for fever.

5. To assess health status of patients in all age groups days **3 and** 7 (active follow up) and up to day 28 (passive follow up) after contact with CHW.

6. To assess management cost of clinical episodes of fever by CHWs with or without RDT's.

7. To study PCR adjusted antimalarial effectiveness of Coartem treatment in children <5 years with uncomplicated malaria by trained CHWs (with or without the diagnostic support of RDT) up to day 42.

8. To explore compliance to Coartem regimen **in all age groups.**

9. To explore the attitudes of CHWs and patients towards RDT's and Coartem at community level.

4. Methods

4.1 General study design:

A community based intervention study with a cross-over design in which Community Health Workers (CHWs) will be trained to distribute Coartem for the management of malaria, with or without the aid of RDT.

4.2 Study area and sample size:

7 villages in Kibaha district with a total population of 8000 people will be included in the study. The whole population represents the study population. A total of 22 CHWs from these villages will be involved in the study. The number of CHWs per village varies between 2 and 4 depending on the total population in the respective village. The geographic distribution of the population has been taken into consideration when determining the number of CHWs for each village. Each CHW will be responsible for between 250 and 400 people in their village. There are 5 dispensaries in the area and clinical officers from these facilities will also be involved. (**See further under 4.4 & 4.6)**

4.3 Sample unit:

Any patient presenting with fever or a history of fever to the CHW for purpose of treatment.

4.4 Specific design:

This is a cross over design in the sense that the same CHW will do malaria diagnosis alternating every week between using RDT and no RDT (clinical diagnosis only) for the entire duration of the study. The study will be performed for 16 weeks without break, preferably covering both high and low transmission seasons.

The Principal Investigator (PI) will ensure that appropriate training relevant to the study is given to CHWs to be able to collect the specimens for further investigation and be well familiarised with the procedure and interpretation of the results of the RDTs. Manufacturer's

instructions and a special training by the study supervisor prior to study initiation will assist the CHW to perform the RDT.

Training at community level will include training of CHW in:

a) Diagnosis of malaria clinically ± RDT
b) Treatment with Coartem, and instructions to patients or parents of its correct intake.
c) Collection of blood samples on filter papers and for blood-smears
d) Information to patients when to come back for follow up
e) Clinical and blood test follow-up, monitoring of compliance and reporting clinical data (including adverse events).

4.5 Inclusion criteria:

a) Fever ("homa") in the last 24 hours according to community-based guidelines prepared by the research team, based on the Integrated management of Childhood Illness (IMCI) algorithm ("simplified IMCI").
b) Informed consent.

4.6 Exclusion criteria:

a) Severe clinical manifestations (according to IMCI algorithm), which require immediate referral to the closest dispensary or the Mlandizi Health Centre.
b) Known or suspected pregnancy - pregnant women will be referred to the dispensary where they will be treated with SP.
c) Previous enrolment within the last 28 days.

4.7 Diagnosis and treatment

Diagnosis and treatment of malaria will be based on clinical assessment + RDT for one week, and on clinical assessment only for the next week, alternating for the entire duration of the study.

The CHWs will have access to Coartem and paracetamol (but not antibiotics). Treatment will be given free of charge to patients enrolled in the study. Coartem is a fixed dose combination of artemether-lumefantrine, which is given in six doses over three days. When a diagnosis of malaria is made, the first dose of the drug will be administered by the CHW (supervised), and the remainder of the drugs in the blister pack will be given to the patient/guardian with

instructions on when and how to take them (unsupervised intake). The patients will be instructed to give locally available fatty foods with the drug, such as coconut-milk, nuts or a teaspoon of cooking oil, but no food will be distributed by the research team.

Coartem treatment is given in standard doses according to body weight as indicated in the table below. ***An alternative method (in accordance with Novartis program) is to choose the correct dose based on the pictures on the Coartem dose chart.***

Weight (kg)	Number of tablets per dose given at 0, 8, 24, 36, 48 and 60 hours	Daily dose of artemether (A) and lumefantrine (L)
5-14	1	40 mg A + 240 mg L
15-24	2	80 mg A + 480 mg L
25-34	3	120 mg A + 720 mg L
>35	4	160 mg A + 960 mg L

4.8 Analysis:

The patients will be divided into 3 age groups for age dependent analyses: 0-4 years, 5-14 years and above 14 years.

4.9 Outcomes

Primary outcome:

Proportion of patients prescribed Coartem (out of all included patients)

Main secondary outcome

Proportion of patients with clinical recovery by day 3 and 7, respectively, out of all included patients.

Other Secondary outcomes:

<u>In all patients</u>

- Proportion of patients prescribed ***antibiotics*** and/or antipyretics (on day 7 patients will be asked if they have been prescribed any other drugs than Coartem from CHW)
- Proportion of patients presenting to CHW within 24 hours after onset of fever (compared to those presenting >24 hours)
- Cost of diagnosis and treatment(s)
- Sensitivity and specificity of RDT vs. microscopy
- Reasons for prescribing Coartem despite negative RDT

- Proportion with new episode of malaria (BS confirmed) within 28 days
- Proportion with Coartem tablets remaining day 3

In children <5 (Coartem – "CCC" study)

- Proportion with new clinical episode of malaria up to day 42
- Proportion with parasitemia/recrudescence (PCR adjusted) day 7 to 42
- Proportion with lumefantrine concentrations below 280 µg/L on day 7±2

Tertiary outcomes

- Attitudes of CHWs and patients towards RDT
- Attitudes of patients to Coartem regimen

4.10 Study Power/sample size estimation

Below is a tentative (very arbitrary) assumption of "fever" and "malaria episode" incidences per month in a population of 1000 people on which to build sample size calculation (assuming 6 months of wet and dry seasons, respectively).

	n	Wet season		Dry season		Yearly fever
		Fever	Malaria	Fever	Malaria	
<5	200	100	50	60	10	960
6-15	300	45	15	30	5	450
≥15	500	50	10	50	5	600
Total	1000	195	75	140	20	1710
Attendance to CHW (80%)	800	156	60	112	16	1368

The sample size/population/number of CHWs suggested by the settled study design:

Population size: 8,000

Number of villages: 7

Number of CHWs: 22

Time period: 16 weeks of patient recruitment + 1 remaining week follow up

Estimated no. of fever patients: Minimum 3,200

Number of malaria infected <5s for Coartem study: minimum 240

The number to be treated with Coartem with/without RDT for 4 months in 8000 people will tentatively be =

Treatment	<5	6-14	≥15	Total
With RDT (assuming 70%, 50% & 30% positivity for respective age group)	1436	481	384	2302
Without RDT (90% for all ages)	1845	865	1153	3863

The available sample size is given in this study. The aim of the following power calculation is to check the influence on the power of potential clustering within the design. Clustering can be assumed on two levels, on the village level and on the CHW level. The latter can be assumed to be potentially the most severe in this study since the outcome of the intervention will be heavily dependent on the behaviour and engagement of the CHWs. In the sequel calculations on village level is omitted potentially resulting in non-conservative results.

The study should be able to detect a reduction of 10 percentage units in Coartem prescriptions in each group when using RDT, i.e. from 80% to 70%, with a power of 80% and a significance level of 5%. Assuming independent data a sample size of 314 fever patients attending the CHW per study group is required which will be met for each age group during the study period. However, under assumption of potential clustering within the 20 CHWs the influence on the power have to be derived. Here, based on the available number of fever cases, table X, the largest possible ICC (Intra Cluster Coefficient) where the power requirements still are fulfilled have been calculated in Table X+1.

Table X. Total sample size per study group (+/- RDT) during study period of 16 weeks in 7 villages with in total 8000 inhabitants.

	<5	6-14	≥15	Total
Total “fever”	1280	600	800	2680
Total “malaria”	480	160	120	760
Fever cases seen by CHW (80%)	1024	480	640	2144

Table X+1. Maximum ICC under the power requirements and given sample sizes for fever cases seen by CHW.

	<5	6-14	≥15	Total
Fever cases seen by CHW (80%)	1024	480	640	2144
Number of cases per CHW	51	24	32	107
Maximum ICC	0.045	0.023	0.033	0.055

From Table X+1 it can be seen that the study design fulfils the power requirements for ICC<0.023 for all age groups. For the total cohort and for children under five years an ICC<0.045, respectively, are possible, which can be regarded as satisfactory since the ICCs in community health setting tend to be small.

It also notable that the study design is paired on a group level, i.e. each CHW is alternately using +/- RDT on weekly basis. This partly controls for the between-CHW variation which reduces the needed sample size compared to ordinary pretest-posttest control group design. The needed sample sizes above can be multiplied with (1-p) where 0<p<1 is the correlation coefficient for random pairs of patients from +/1 RDT groups treated by the same CHW. Assuming p=0.10 will then decrease the needed number sample size to 90% out of the given figures.

4.11 Study products

Paracheck Pf manufactured by Orchid Biomedical Systems, India, will be used as RDT. This test has been chosen for the study because it is the one that has been identified by the MOH as the diagnostic test to be used in directing treatment with Coartem in areas where microscopy is not available. This test has been evaluated in other areas and found to be highly sensitive and specific, with rates of >95% and 100%, respectively (Dev, 2004; Huong et al., 2002). The tests have been purchased from Biocare, Dar es Salaam. CHWs will be trained on how to perform the tests according to manufacturer's instructions.

Coartem® (Novartis, Switzerland) will be used for treatment. Coartem was purchased from the retailer (Heko Pharmaceuticals, Dar es Salaam) and repacked from packages of 2 x 8 tablets. Blisters were cut and repacked into specially developed and colour-coded packages for each patient group as determined by patient weight. The drug will be given in 6 doses as described by the manufacturer. CHWs will similarly be trained on how to administer the dose

according to manufacturer's instructions. Only the first dose will be given under supervision (of CHW) (see also 4.7).

4.12 Study sites

The study will be conducted in 7 villages with 5 dispensaries. During preparatory visits to the study area, the following villages have been identified: Kikongo, Mwanabwito, Ngeta, Mbwawa Shule, Mbwawa Mkoleni, Miswe Juu and Miswe Chini.

Kibaha district is one of two districts in Coast region where the Muhimbili University College of Health Sciences/Karolinska Institutet (MUCHS/KI) Malaria consortium has carried out an intervention study aimed at improving malaria case management in children <5 years at Primary Health Care (PHC) facilities.

For many years, each village in Kibaha district has had two CHWs. An additional number of village volunteers have been trained previously in an IMCI programme for reinforcing and monitoring good health practices in the community to make a total of 20 CHWs. The project will select CHWs for the study from the pool already trained health volunteers in each village. In the selection process, district authorities and supervisors, clinical officers serving at the dispensaries and village leaders will assist in a structured way in suggesting names of well-functioning CHWs in the village.

4.13 Enrolment of patients

Patients will be enrolled when they present to the CHW with fever or history of fever. All patients, male and female, presenting at any CHW involved in the study with symptoms of fever in the preceding 24 hours will be screened for eligibility. If they fulfil the inclusion criteria and do not fulfil any exclusion criteria they will be enrolled in the study. All enrolled patients will receive a unique study code, consisting of 6 digits where the first two are unique for the CHW, and the next four is the patient's serial number, and the last two show the dose of Coartem given.

The study enrolment will be conducted for a total of 16 weeks including both high and low transmission periods.

After diagnostic judgement ±RDT, the patients will receive treatment with Coartem if considered in need of malaria treatment.

4.14 Follow-up

Patients will be advised to come back on day 3 and 7 (active follow up). Apart from this mandatory follow up, patients will be counselled to come back in case of new fever between day 7 and 28 (passive follow up).
For special follow-up of <5 children in "CCC" study, see separate protocol.

4.15 Randomisation

Each site will be randomly allocated to either start to use or not to use RTD during the initial study week, after which they alternately will change between RDT aided/unaided malaria diagnosis every week. The RDTs will be numbered and the number filled out on the forms. The same CHW will thus perform clinical and RDT aided diagnoses so as to minimise user dependency. Neighbouring villages will not be synchronised.

4.16 Concomitant drug therapy

Substance and day of administration for any antibiotics or other treatments (including traditional remedies) taken during the first 7 days will be recorded in the CRF on day 7.

4.17 Collection of blood samples and performance of laboratory tests

At enrolment all patients will have blood tests taken from finger pricks. A few drops of blood will be collected for RDT and a blood smear for later microscopical examination at central level. For children <5 years ("CCC" study) circa 100 µl of blood will also be collected on filter paper (FP). Each sample (RDT, FP and blood slide) will be labelled by using a sharpened pencil and will contain the following information: identity with code and name and date of sampling.

RDTs will be performed on the spot by the CHW, and results will be used to guide treatment. The collected blood smears will be stored in special boxes and picked up three times a week by the field supervisors, and brought to the research laboratory in Mlandizi. In Mlandizi they will be stained with 3% Giemsa for 30 minutes, and standard microscopy examination will be done. Asexual parasite density will be calculated by counting parasites against 200 white

blood cells (wbc), assuming the wbc of 8000/µl. If less than 10 parasites are detected per 200 wbc, estimates will be made against 500 wbc.

Quality control by backup microscopy (5% in MUCHS and 5% in KI) will be done for 10% of blood slides by blinded and independent microscopists.

For “CCC” study children only = blood samples on FP for PCR analysis and an additional FP for choloroquine/SP analysis (FP_2) as well as whole blood samples in tubes for determination of lumefantrine concentration by High Performance Liquid Chromatography (HPLC) (L conc) will be collected on the days indicated in the flow chart and according to methodologies described in Coartem effectiveness protocol. Analyses of these samples will be done at the Malaria Research Unit, KI, for PCR, and for lumefantrine concentrations (HPLC) at the research unit of Y. Bergqvist in Falun, Sweden. For SP and chloroquine analyses will be done at MUCHS if capacity in place or in Falun, Sweden.
The methodologies for collection of “FP_2 “ and “L conc” samples are elaborated in “CCC” study protocol. Briefly blood samples of 50 µl (or 100 µl) for FP_2 will be collected on Whatman 3MM filter paper. The L conc in heparinised capillary tubes with minimal amount of 100 µl blood.

4.18 Follow-up (See flow chart)**:**
A: for >5 years old
Day 0: questionnaire, blood smear, RDT
Day 3: questionnaire
Day 7: questionnaire, blood smear
Day 8 – 27 (upon fever): blood smear

B: For <5 years old (for further details see separate protocol)
Day 0: questionnaire, blood smear, RDT + FP_1
Day 3: questionnaire + L conc
Day 7: questionnaire, blood smear + L conc.
Day 8 – 27 (upon fever): blood smear + FP_1
Day 28: questionnaire, blood smear + FP_1
Day 42: questionnaire, blood smear + FP_1

All persons entering the study will be given a study card. The CHW will note names of all patients seeking care in a record book. The identity card will have the following information: Name of person, age, name of CHW, any previous serious adverse reaction, and date of each entry in the study since repeated entries may occur. The information in the study will be written on the questionnaires that will have the same identification number.

During the study the research team will visit each CHW three days every week to collect data, monitor performance and to re-supply drugs, RDTs and material as required. The villages will have overlapping starting days, some on a Tuesday, some on a Wednesday for practical reasons. The villages included in "CCC" study will be visited every day of the week.

Patients who come back with fever between days 8-28 will have a blood slide taken, and referred to the village dispensary for further investigation and management.

4.19 An Integrated Referral System

Symptoms of severe malaria are exclusion criteria for this study and the CHWs will be instructed to promptly refer patients with coma or lethargy, convulsions, severe difficulty in breathing, constant vomiting or inability to drink or breastfeed to the facility appropriate depending on time and day. The village dispensaries are open daytime on weekdays whereas the Mlandizi Health Centre has 24 hours service. The distance from dispensaries to the Mlandizi Health Centre varies between 9 and 18 km.

Training on the recognition of severe malaria will be given during the training course where severe malaria will be defined according to the existing guidelines (National Malaria Control, Ministry of Health). Health workers in the public health sectors will be informed about the study and thus interact with CHWs.

4.20 CHW follow-up questionnaire interviews

A follow-up evaluation of CHW experience of RDTs and ACTs will be done in a rather brief and restricted manner.

One question will be the extent and reasons for prescribing Coartem despite negative RDT.

In addition, the attitudes of CHWs and patients generally towards to RDTs as diagnostic tools as well as the attitudes to Coartem packaging and regiments will be explored.

The use of (insecticide treated) bed nets will also be explored.

Tentative focus group contents/methodology to be developed.

(To be developed)

4.21 Monitoring/supervision

A plan for monitoring of study sites will be prepared. The PIs, co-investigator(s) and field supervisors will ensure that the facilities for conducting the tests remain acceptable and will ensure the results of the study are being recorded accurately in the CRFs.

The research team will meet each CHW 3 times a week.

4.22 Study registration

The study will be registered at www.clinicaltrials.com before enrolment of the first patient.

4.23 Data Management and evaluation

Data will be entered and managed in a secure computer environment at MUCHS. The registry for data entry will be = EPI-data . Any corrections or discrepancies will be recorded. Primary analysis will be performed at MUCHS but major and final analysis will be at KI. **Final analysis program yet to be determined.**

4.24 Data dissemination and publication

The right to publish and disseminate information derived from the performance of the work will be decided upon jointly by KI and MUCHS. Qualification for authorship shall be in keeping with generally accepted criteria, i.e. having provided significant contribution to the study.

Publications shall carry appropriate acknowledgement of funding support, while authors maintain responsibility for the content.

4.25 Ethical considerations

The study will be performed in accordance with Good Clinical Practice (GCP) and the principles stated in the declaration of Helsinki. Ethical clearance will be sought from the appropriate ethics committees of MUCHS/TZ and in Sweden, before enrolment of any subjects into the study. Copies of the clearances from respective committee are provided in Appendix 6. The opinions of the committees should be dated and in writing. The PIs and Study Director are responsible for informing the respective committee of any amendments to the protocol.

The investigator will ensure that each subject or parent/guardian is given thorough verbal and written information about the nature, purpose and possible risks of the study. The subjects and/or parents/guardians will be informed that they are free to discontinue their participation in the study at any time. Informed consent will be obtained at two levels: at village level, and at individual level. At the individual level, the informed consent will be administered by the trained CHW at the time of enrolment. Each informed consent will be recorded by the PI. Written and verbal information will be given in Kiswahili.

The cross over design (every week) will be dependent on time, but will continue for 16 weeks or less if the sample size is achieved. We anticipate no objection in withdrawing the RDT after every week. Every febrile case will have access to treatment with antipyretics. In the case of RDT negative or when not done, the results of the BS will be available only several days after initial treatment has been issued. The BS results will not influence treatment. Follow up visits will not have RDT but a blood slide will be taken in case of fever.

No change in the investigational procedures shall be affected without the mutual agreement of the study investigators. All changes must be documented by signed protocol amendments.

It could be argued that this study empowering the villages with ACT will weaken health facilities that will not provide ACT during the study period. This issue will be dealt with by comprehensive information to both communities and health facilities.

4.26 Ensuring an effective and sustainable study drug supply system

The study drugs and RDTs will be stored at MUCHS, from where deliveries will be made to the study district by the central research team. The field manager will ensure delivery of drugs and RDTs to the health facilities in the study areas. The field manager will be responsible for storing the drugs and RDTs at the clinic.

Enrolled patients will obtain study drugs from the CHWs. The field supervisors will maintain records of the drugs received, distributed and balance in stock. The RDTs will be numbered in series for correct identification. Appropriate storage containers will be provided to CHWs and study supervisors.

5. Capacity strengthening

5.1 Community level

The study will link to the existing health structure in Tanzania, using the CHW and dispensaries and will result in capacity building at community level with:

- Skilled and motivated CHWs who perform well with regards to malaria symptom recognition, handling and reading of the RDTs, dispensing of treatment, recognition of severe illness and advise on home and referral care. The IMCI guidelines for children will be reinforced in the training and monitoring of CHWs.
- A continuous and uninterrupted supply of drugs for treatment of malaria within the community and at the local health units.
- Increased awareness among CHWs of the need to refer those suffering from conditions not targeted by the programme and those severely ill to higher levels of care.
- A system for support supervision and retraining of the community CHWs.
- Well informed communities within which caretakers of children < 5 years have knowledge and skills for prompt recognition of fever, early appropriate care seeking, prompt administration of efficacious drugs, complete adherence to treatment dosing schedules, early identification of severe illness, complications or lack of improvement and prompt referral.

In order to effectively address the above concerns, the key components of the interventions will be:

- Training and support for CHWs
- An efficient and sustainable study drugs supply system

- An integrated referral and monitoring system

5.2 Field Research Team

The study will locally recruit and further develop a field research team linked to MUCHS for the present and future studies in public health and epidemiology.

5.3 Research and Training Programme

The study is part of Research and Training PhD Programmes for M. Mubi (RDT part) as well as B. Ngasala and A. Mårtensson (Coartem part). A. Mårtensson has attended a research course in clinical trial and GCP. This is also planned for B. Ngasala. The study may represent a starting point for development of Ph D programme for Gloria Maganga.

5.4 Laboratory technology transfer

A technician, if identified, may be trained in PCR technology at KI. Lumefantrine concentration assay may be considered for MUCHS after further development and standardisation in Sweden (Y. Bergqvist).

6. Study Components and Time Plan

6.1 June 15th – December 15th *(Ongoing activities at different levels of implementation)*

1. Finalize this RapAct study plan and seek approval with all contributors
2. Prepare a budget for the RapAct study
3. Prepare plan of study implementation
4. Apply for consent with the two involved ethical committees (MUCHS and KI)
5. Establish ways of securing access to ACT throughout the study
6. Establish ways of securing access to RDT throughout the study
7. Identify teaching materials, IMCI manuals & develop a training manual for the study
8. Identify a lab technician, field supervisors and data managers for a field office
9. Establish access to all needed equipment and laboratory consumables: slides, special filter papers etc
10. Develop identification cards
11. Develop means for computer storage of data
12. Develop CRFs and questionnaires

6.2 Preparations in the field: Ongoing activity started in August 2005

1. Establish knowledge of and relations with the leaders of the health structure in the area, and introduce the project. The objectives of the studies, the benefits and possible side effects will be explained. The existing community sources of treatment for malaria will be explored.
2. Employ and train field supervisor. Their tasks should be:
   i. Monitor that CHWs have all supplies all the time.
   ii. Collect all record forms and samples from each CHW three times a week.
   iii. Monitor the CHWs to ensure that they are keeping good records of patients treated and drugs received.
   iv. Monitor the CHW to check that they are using RDT on correct weks only.
   v. Inform the Coordinator on activities in the community.
3. Identify Community health workers to be involved in the study through identification by the communities (in collaboration with the District IMCI coordinator and village leaders). The qualities of CHWs suitable for the study should be:
   i. A permanent resident of the community.
   ii. Able to read and write.
   iii. Respected member of the community.
   iv. A good community mobiliser.
   v. Able to devote time and effort to the study.
   vi. Living in a house that is geographically well located in the village and in the relation to other involved CHWs.
4. Get to know the referral systems
5. Test the teaching materials

6.3 Teaching period (*20th - 24th February 2006*)

1. Training of the chosen CHWs (and Clinical officers) for one week.
2. The following messages will be emphasised in IEC:
   i. Prompt recognition of malaria symptoms.
   ii. Prompt treatment and compliance with study drugs.
   iii. Importance of fat intake with ACT
   iv. Prompt recognition of non-response to treatment and the need to promptly seek treatment from a trained health facility worker.

v. Prompt recognition of severe disease and IMCI danger signs in children and prompt referral to appropriate level.
vi. The need to comply with referral for severe disease and non-response to initial treatment
vii. Practical skills in performing tests of blood and storage of samples

6.4 Intervention phase (March to June 2006)

6.5 PCR and drug analyses (For details – see Coartem study proposal)

6.6 Data analysis (To be determined) will be performed mainly in Stockholm.

6.7 Inputs of Technical Advisors and Collaborators

Karin Kallander: RDT study design, qualitative data analysis
Max Petzold: Statistical Analysis
Andreas Mårtensson: Coartem study design, MSP genotyping
Yngve Bergqvist: Lumefantrine concentration analysis
Pedro Gil: Molecular study design
Christin Sisowath: Drug resistance/tolerance genotyping

6.8 Field Research Team (see Appendix 1)

7. Time plan:

Month / Task	May 2005	Jun	Jul	Aug	Sep	Oct	Nov	Dec	Jan 2006	Feb	Mar	Apr	May	Jun	Jul	Aug – Oct 06
Information	√															
Field Prep				√	√	√	√									
Finalisation of proposal & other study arrangements								√	√							
Teaching										√						
Intervention											√	√	√	√		
PCR + Drug															√	√

analyses																
Data entry and analysis											√	√	√	√	√	√

8. Budget

8.1 RapAct budget in Tanzania (Appendix 2)

8.1.1. Development of HQ in Mlandizi

8.1.2 Workshop 20-24, February 2006

8.1.3 Master budget

8.2 RapAct budget in Sweden

Flow chart for RapAct study

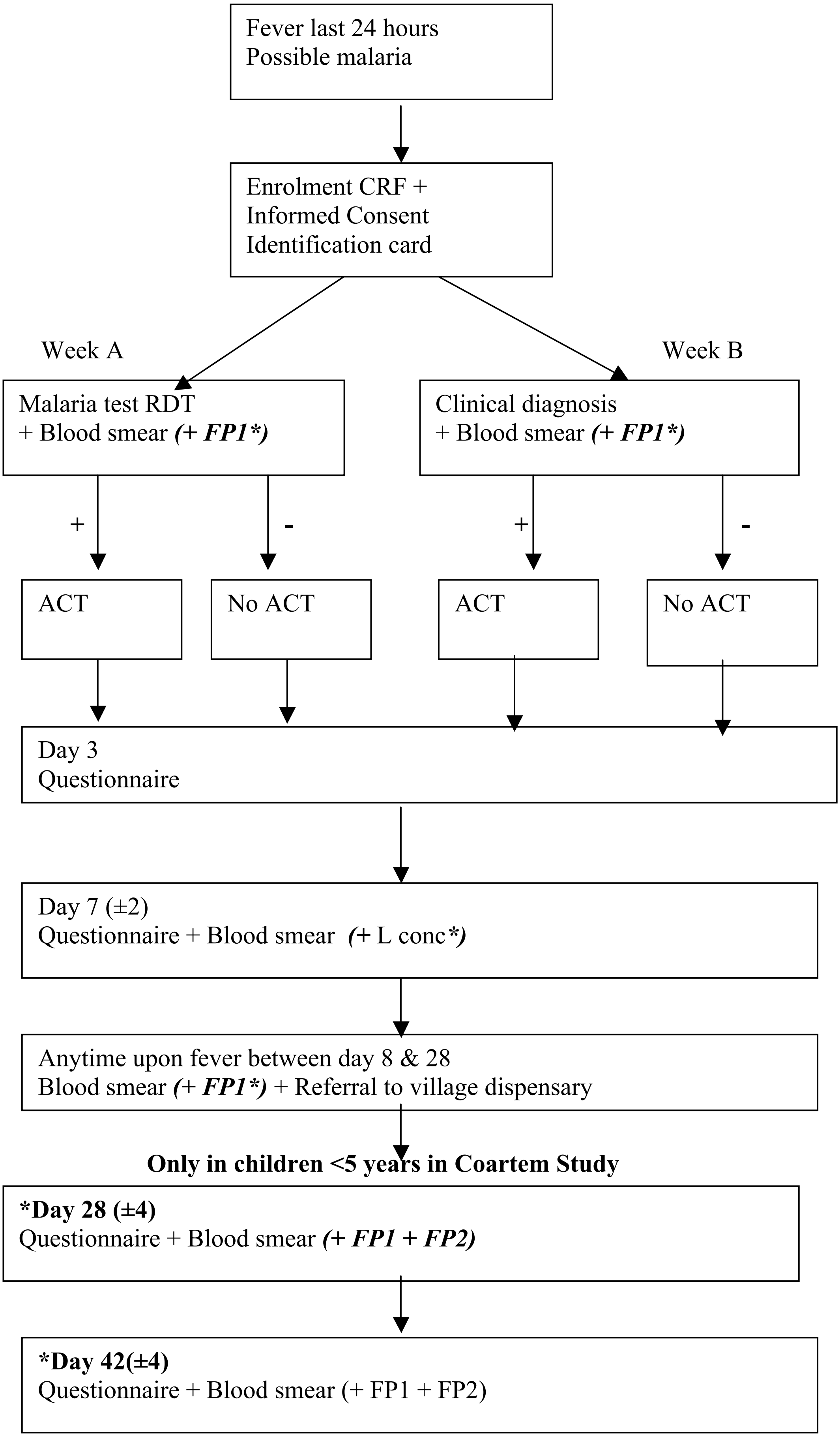

*Only in children <5 years in Coartem effectiveness study ("CCC")

FP1 = Field paper for parasite genotyping
FP2 = Field paper for CQ/SP concentration assay

9. References:

Arora S., Gaiha M. & Arora A. (2003) Role of ParaSight-F test in the diagnosis of complicated *Plasmodium falciparum* malarial infection. *Brazilian Journal of Infectious Diseases*, **7(5):** 332-338.

Arrow K.J., Panosian C. & Gelband H. (Eds) *Saving Lives, Buying Time: Economics of Malaria Drugs in an Age of Resistance.* Board on Global Health, National Academy Press, 2004.

Aslan G., Ulukanligil M., Seyrek A. & Erel O. (2001) Diagnostic performance characteristics of rapid dipstick test for *Plasmodium vivax* malaria. *Mem Inst Oswaldo Cruz, Rio de Janeiro*, **96(5)**: 683-686

Bell D., Go R., Miguel C., Walker J., Cacal L & Saul A. (2001) Diagnosis of malaria in a remote area of the Philippines: comparison of techniques and their acceptance by health workers and the community. *Bull WHO*, **79(10):** 933-941.

Björkman A. & Bhattarai A. (2005) Public Health Impact of drug resistant *Plasmodium falciparum* malaria. *Acta Tropica*. 2005 Jun **94(3):** 163-169.

Hastings M.D., Bates S.J., Blackstone E.A., Monks S.M., Mutabingwa T.K. & Sibley C.H. (2002) High pyrimethamine-resistant alleles of dihydrofolate reductase in isolates of *Plasmodium falciparum* from Tanzania. *Trans R Soc Trop Med Hyg*, 96**(6)**: 674-6

Kallander K., Nsungwa-Sabiiti J., Peterson S. (2004) Symptom overlap for malaria and pneumonia – policy implications for home management. *Acta Tropica*, **90(2)**: 211-4

Mkandala C., Marum L., Kamila N., Chizani N. & Mhango A. (2000) Implications of Integrated Management of Childhood Illnesses (IMCI) on sulfonamide and other antibiotic usage for malaria, anaemia and respiratory infections in Blantyre, Malawi. *Am J Trop Med Hyg* **62** Suppl: 137-138.

Moody A. (2002) Rapid diagnostic tests for malaria parasites. *Clinical Microbiology Reviews*, June 2002: 66-78.

Nsimba SED (2003) Exploring malaria case management of underfive children in households and public primary health care facilities in Kibaha district, Tanzania. PhD thesis. Karolinska Institute, Sweden.

O'Dempsey T. J. D., McArdle T. F., Laurence B. E., Lamont A. C., Todd J. E. & Greenwood B. M. (1993) Overlap in the clinical features of pneumonia and malaria in African children. *Trans R Soc Trop Med Hyg* **87**: 662-665.

Piola P., Fogg C., Bajunirwe F. et.al. (2005) Supervised versus unsupervised intake of six-dose artemether-lumefantrine for treatment of acute, uncomplicated *Plasmodium falciparum* malaria in Mbarara, Uganda: a randomised trial. *Lancet*, **365**:1467-73

Rooth I and Björkman A. (1992). Fever episodes in a holoendemic area of Tanzania. Parasitological and clinical findings and diagnostic aspects related to malaria. Transactions of the Royal Society of Tropical Medicine and Hygiene 86:479-482, 1992.

Singh N. & Saxena A. (2005) Usefulness of a rapid on-site *Plasmodium falciparum* diagnosis (Paracheck PF) in forest migrants and among indigenous population at the site of their occupational activities in Central India. *Am. J. Trop Med Hyg*, **72(1)**: 26-29

Singh N. & Shukla M. M. (2002) Short report: Field evaluation of post treatment sensitivity for monitoring parasite clearance of Plasmodium falciparum malaria by use of the DETERMINE malaria Pf test in Central India. *Am. J. Trop. Med. Hyg*., **66(3)**: 314-316.

Singh N., Saxena A. & Sharma V.P. (2002) Usefulness of inexpensive, Paracheck test in detecting asymptomatic infectious reservoir of *Plasmodium falciparum* during dry season in inaccessible terrain in Central India. *Journal of Infection*, **45**: 165-168

Tjitra E., Suprianto S., McBroom J., Currie B. J. & Anstey N. M. (2001) Persistent ICT Malaria P.f/P.v Panmalaria and HRP2 antigen reactivity after treatment of *Plasmodium falciparum* malaria is associated with gametocytemia and results in false-positive diagnoses of *Plasmodium vivax* in convalescence. *Journal of Clinical Microbiology*, March 2001; 1025-1031.

WHO (2000) New Perspectives. Malaria Diagnosis. Report of a joint WHO/USAID Informal Consultation 25-27 October 1999. World Health Organisation, Geneva, Switzerland. WHO/MAL/2000.1091.

WHO/UNICEF (2003) The Africa Malaria Report 2003. WHO/CDC/MAL/2003.1093

Wongsrichanalai C. (2001) Rapid diagnostic techniques for malaria control. *Trends in Parasitology*, **17(7)**: 307-309.
